# Cost-effectiveness of Prostate Radiation Therapy for Men With Newly Diagnosed Low-Burden Metastatic Prostate Cancer

**DOI:** 10.1001/jamanetworkopen.2020.33787

**Published:** 2021-01-13

**Authors:** Nataniel H. Lester-Coll, Steven Ades, James B. Yu, Adam Atherly, H. James Wallace, Brian L. Sprague

**Affiliations:** 1 Division of Radiation Oncology, University of Vermont Larner College of Medicine, Burlington; 2 University of Vermont Cancer Center, Burlington; 3 Division of Hematology and Oncology, University of Vermont Larner College of Medicine, Burlington; 4 Department of Therapeutic Radiology, Yale School of Medicine, New Haven, Connecticut; 5 Center for Health Services Research Department of Medicine, University of Vermont Larner College of Medicine, Burlington; 6 Department of Surgery, University of Vermont Larner College of Medicine, Burlington

## Abstract

**Importance:**

Prostate radiation therapy (PRT) is a treatment option in men with low-volume metastatic prostate cancer based on the results of the Systemic Therapy in Advancing or Metastatic Prostate Cancer: Evaluation of Drug Efficacy Arm H (STAMPEDE-H) trial. However, the cost-effectiveness of this treatment remains unaddressed.

**Objective:**

To assess the cost-effectiveness of PRT when added to androgen deprivation therapy (ADT) for men with low-volume metastatic hormone-sensitive prostate cancer (mHSPC).

**Design, Setting, and Participants:**

This economic evaluation used microsimulation modeling to evaluate the cost-effectiveness of adding PRT to ADT. A simulated cohort of 10 000 individuals with low-volume mHSPC was created. Data from men with low-volume mHSPC were extracted and analyzed from January 18, 2019, through July 4, 2020. Transition probabilities were extracted from the STAMPEDE-H study. Health states included stable disease, progression, second progression, and death. Individual grade 2 or higher genitourinary and gastrointestinal toxic events associated with PRT were tracked. Univariable deterministic and probabilistic sensitivity analyses explored uncertainty with regard to the model assumptions. Health state utility estimates were based on the published literature.

**Exposures:**

The combination of PRT and ADT using regimens of 20 fractions and 6 weekly fractions.

**Main Outcomes and Measures:**

Outcomes included net quality-adjusted life-years (QALYs), costs in US dollars, and incremental cost-effectiveness ratios. A strategy was classified as dominant if it was associated with higher QALYs at lower costs than the alternative and dominated if it was associated with fewer QALYs at higher costs than the alternative.

**Results:**

For the base case scenario of men 68 years of age with low-volume mHSPC, the modeled outcomes were similar to the target clinical data for overall survival, failure-free survival, and rates of PRT-related toxic effects. The addition of PRT was a dominant strategy compared with ADT alone, with a gain of 0.16 QALYs (95% CI, 0.15-0.17 QALYs) and a reduction in net costs by $19 472 (95% CI, $23 096-$37 362) at 37 months of follow-up and a gain of 0.81 QALYs (95% CI, 0.73-0.89 QALYs) and savings of $30 229 (95% CI, $23 096-$37 362) with lifetime follow-up.

**Conclusions and Relevance:**

In the economic evaluation, PRT was a dominant treatment strategy compared with ADT alone. These findings suggest that addition of PRT to ADT is a cost-effective treatment for men with low-volume mHSPC.

## Key Points

**Question:**

In men with newly diagnosed, low-volume metastatic prostate cancer, is the addition of prostate radiation therapy to androgen deprivation therapy cost-effective?

**Findings:**

In this economic evaluation using data from a simulated cohort of 10 000 individuals with low-volume metastatic prostate cancer, a microsimulation model found that the addition of prostate radiation therapy to standard-of-care androgen deprivation therapy was associated with reduced net costs and improved quality-adjusted life-years and was therefore a dominant cost-effective strategy.

**Meaning:**

These findings support the incorporation of prostate radiation therapy as part of initial treatment for men with low-volume metastatic prostate cancer.

## Teaser

This economic evaluation assesses the cost-effectiveness of the addition of prostate radiation therapy to androgen deprivation therapy for men with low-volume metastatic hormone-sensitive prostate cancer.

## Introduction

Prostate cancer is the most common malignant tumor diagnosed in men.^1^ Metastatic prostate cancer is diagnosed in 3% of men, and up to 10% of men with initial localized disease develop distant metastases.^2,3^ The management of hormone-sensitive metastatic prostate cancer (mHSPC) is complex, with multiple recent randomized clinical trials^4,5,6,7,8,9^ demonstrating improvements in cancer outcomes with the addition of advanced systemic agents to androgen deprivation therapy (ADT). A recent innovation in the management of mHSPC is the addition of prostate radiation therapy (PRT) for men with newly diagnosed mHSPC with low metastatic burden.

The Systemic Therapy in Advancing or Metastatic Prostate Cancer: Evaluation of Drug Efficacy Arm H (STAMPEDE-H) trial examined the impact of adding PRT to ADT in men with newly diagnosed mHSPC.^10^ With a median follow-up of 37 months, the trial found that PRT improved failure-free survival (FFS) but not overall survival (OS) in unselected patients. However, in men with low-volume disease, PRT improved both FFS and OS. In response to the publication of that trial, PRT is now considered a standard-of-care treatment for men with low-volume mHSPC in the US according to the National Cancer Center Network.^11^ However, PRT introduces additional costs and toxic effects that are not accounted for when OS and FFS are examined in isolation.

For wide implementation of PRT and insurance coverage, cost-effectiveness analysis (CEA) is essential. Health care costs, including expenditures for prostate cancer, are increasing in the US, and there is increasing emphasis on providing value-based care.^12,13^ Thus, evaluating the cost-effectiveness of new therapies and projecting their potential financial consequences are instrumental in determining the appropriateness for widespread use. The objective of this study was to perform a CEA to assess the value of adding PRT to ADT for men with low-burden, newly diagnosed mHSPC.

## Methods

For this economic evaluation, we developed a microsimulation model using TreeAge Pro 2020 software (TreeAge Software LLC) to estimate the cost-effectiveness of adding PRT to standard-of-care ADT among men with mHSPC using model inputs based on published literature. Data from men with low-volume mHSPC were extracted and analyzed from January 18, 2019, through July 4, 2020. Our methods conformed to the Society for Medical Decision Making best practice guidelines for model transparency and validation.^14,15^ This economic evaluation used no individual patient-level data to inform the model. Therefore, it does not constitute human subjects research and does not require institutional review board review or exemption according to the US Department of Health and Human Services (45 CFR §46). This study followed the Consolidated Health Economic Evaluation Reporting Standards (CHEERS) reporting guideline.

### Patients and Treatment

Clinical data were extracted from the STAMPEDE-H trial.^10^ This multicenter randomized clinical trial assessed 2061 men with newly diagnosed metastatic prostate cancer who were randomized to receive standard-of-care ADT with or without PRT. Prostate radiation therapy was 55 Gy in 20 daily fractions of 2.75 Gy for 4 weeks or 36 Gy in 6 consecutive weekly fractions of 6 Gy. Among 819 patients with low-metastatic burden (≤4 bony sites and the absence of visceral metastases), OS was improved using either fractionation regimen (HR, 0.68; 95% CI, 0.52–0.90; *P* = .007). Three-year survival was 73% in the control group vs 81% in the PRT group.

### Microsimulation Model

**Figure 1.**
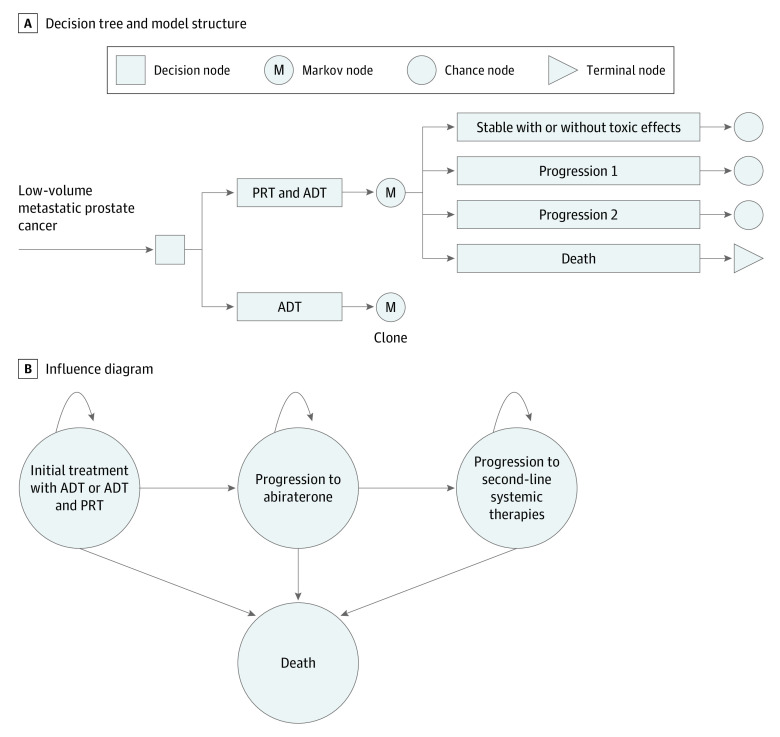
Microsimulation Model A, Abbreviated decision tree and model structure used to compare 2 strategies for treating low-volume metastatic prostate cancer. Progression 1 indicates progression after initial treatment (androgen deprivation therapy [ADT] plus prostate radiation therapy [PRT] or ADT). Progression 2 indicates progression after abiraterone treatment. B, Influence diagram showing the network of 4 disease-related health states. M indicates Markov model.

We used the microsimulation model to perform a CEA of ADT vs ADT plus PRT from a US payer perspective. The health states were stable disease after initial treatment, progression, second progression, and death (Figure 1). Patients received abiraterone at first progression and additional systemic therapies for castration-resistant disease at second progression (docetaxel, enzalutamide, cabazitaxel, sipuleucel-T, and radium 223 dichloride).^16^ Cost-effectiveness was assessed by calculating discounted quality-adjusted life-years (QALYs), cumulative costs, and the incremental cost-effectiveness ratio. A treatment strategy with an incremental cost-effectiveness ratio less than the societal willingness to pay was considered to be cost-effective. In the base case, willingness to pay was defined as $100 000 per QALY gained.^17^ A strategy was classified as dominant if it was associated with higher QALYs at lower costs than the alternative and dominated if it was associated with fewer QALYs at higher costs than the alternative. The model was run with 10 000 individual trials, and stochastic uncertainty was reported as the 95% CIs around the mean model estimates.

The time horizon was 37 months to mirror the median follow-up in the STAMPEDE-H trial. The model was then run until all patients died to explore how the additional simulated follow-up time and events affected the model’s results. An advantage of a microsimulation model over a state-transition Markov model is the ability to model and track individual events, such as treatment-related toxic effects. Grade 2 or higher genitourinary and gastrointestinal toxic effects were followed in the model using trackers with individual Monte Carlo simulations.

### Transition Probabilities

All model inputs are summarized in Table 1. Transition probabilities were calculated from the STAMPEDE-H trial. Data were graphically extracted from the published Kaplan-Meier curves using a validated graphical digitizer (WebPlotDigitizer, version 4.2; Ankit Rohatgi, MD, MBA). Cycle-specific transition probabilities were calibrated to the STAMPEDE-H data using an iterative, nonlinear optimizing algorithm to minimize the difference between the target data (STAMPEDE-H) and the modeled data. For the probabilistic sensitivity analysis, uncertainty in the transition probabilities was modeled using β distributions (which are bounded by 0 and 1) based on the interquartile range.^22^ Uncertainty in the estimates of the hazard ratios (HRs) were modeled by γ distributions, which are bounded by 0 and infinity.^23^

**Table 1.** Model Parameters and Assumptions

Variable	Base case and modeled distribution (range)	Reference
**Transition probability, β distributed**		
Progression from stable state		
ADT plus PRT	0.020 (0.015-0.025)	Parker et al, 2018^10^
ADT	0.035 (0.026-0.044)	Parker et al, 2018^10^
Death		
During stable state	0.0001 (0.0008-0.0013)	Parker et al, 2018^10^
During progression state	0.050 (0.038-0.062)	Parker et al, 2018^10^
Probability of grade ≥2 toxic effects		
Genitourinary tract	0.003 (0.002-0.004)	Dearnaley et al, 2016^18^
Gastrointestinal tract	0.003 (0.002-0.004)	Dearnaley et al, 2016^18^
Hazard ratio for progression to PRT, γ distributed	0.59 (0.25-1.00)	Parker et al, 2018^10^
**Utilities, β distributed**		
ADT plus PRT	0.90 (0.78-0.98)	Stewart et al, 2005^19^
PRT	0.83 (0.71-0.91)	Stewart et al, 2005^19^
Toxic effects associated with PRT		
Gastrointestinal tract	0.79 (0.61-0.90)	Stewart et al, 2005^19^
Genitourinary tract	0.90 (0.78-0.98)	Stewart et al, 2005^19^
Genitourinary and gastrointestinal tracts	0.76 (0.48-0.88)	Stewart et al, 2005^19^
Progression 1^a^	0.70 (0.56-0.84)	Stewart et al, 2005^19^
Progression 2^b^	0.11 (0.01-0.52)	Stewart et al, 2005^19^
**Costs, γ distributed, $**		
ADT plus PRT once	16 860 (7243-39 628)^c^	Institutional Medicare fees
ADT monthly	63 (47-79)	Pollard et al, 2017^16^
Progression 1^a^	5738 (4244-7252)	Pollard et al, 2017^16^
Progression 2^b^	17 365 (13 023-21 707)	Pollard et al, 2017^16^
Death once	5772 (4617-6926)	Obermeyer et al, 2014^20^
Toxic effects associated with radiation therapy once	2208 (1766-2649)	Pan et al, 2018^21^

Abbreviations: ADT, androgen deprivation therapy; PRT, prostate radiation therapy.

^a^ Progression 1 is progression after initial treatment (ADT plus PRT or ADT) (monthly abiraterone treatment).

^b^ Progression 2 is progression after abiraterone treatment (monthly second-line systemic therapy).

^b^ Base case estimate based on 20 fractions. Range includes 6 weekly fractions to 44 daily fractions.

### Costs and Utilities

This analysis was conducted from a US payer perspective. Costs reflected Medicare rates with the exception of costs related to PRT-related toxic effects. We used institutional Medicare fees for the cost of PRT (eTable 1 in the Supplement). Costs of leuprolide acetate, abiraterone, and other systemic therapies were taken from a study that detailed billing data and Medicare reimbursement for these drugs.^16^ A weighted mean was used for systemic therapies for castration-resistant disease after first progression. Cost estimates for toxic effects were derived from a nationwide sample of private claims data of intensity-modulated radiotherapy for prostate cancer using the mean complication costs.^21^ This sample was the only source of private insurance claims in our study, and it was chosen because it represents the most comprehensive US cost analysis of complications after intensity-modulated radiotherapy, to our knowledge. Costs of end-of-life care were from a national analysis of Medicare beneficiaries with cancer with a poor prognosis.^20^ Costs were discounted at a 3% annual rate to adjust for inflation and were adjusted to 2020 US dollars using the Consumer Price Index.^15,24^

Because robust, long-term toxicity data were not available for radiotherapy doses used in the STAMPEDE-H trial, grade 2 or higher gastrointestinal and genitourinary toxic effects associated with PRT were based on the Conventional or Hypofractionated High-dose Intensity Modulated Radiotherapy In Prostate Cancer (CHHiP) trial, which used a slightly higher radiation dose of 60 Gy in 20 fractions.^18^ Health state utilities or patient preferences were based on a sample of men 60 years or older, half of whom had prostate cancer, using the standard gamble method.^19^

### Statistical Analysis

Deterministic sensitivity analyses were conducted for all parameters to evaluate the extent to which uncertainty and variability influenced model results. We studied ranges that corresponded to the reported 95% CI or interquartile range around a parameter and used whichever was largest. The base case of our model was calibrated to the STAMPEDE-H clinical trial in which patients received leuprolide acetate as standard of care. Given that therapies such as abiraterone are now being used for mHSPC based on the Long-Acting Therapy to Improve Treatment Success in Daily Life (LATITUDE) and STAMPEDE-G studies,^6,7^ we performed an additional analysis of the addition of abiraterone in the upfront setting to ADT and PRT. In addition, probabilistic sensitivity analyses using 10 000 Monte Carlo simulations while sampling with replacement from distributions of all input parameter inputs were performed to explore uncertainty with regard to the transition probabilities, costs, and utilities.

## Results 

For the base case scenario of men 68 years of age with low-volume mHSPC, the modeled outcomes were similar to the target clinical data with regard to OS, FFS, and rates of PRT-related toxic effects (eTable 2 and eFigure 1 in the Supplement). With 37 months of follow-up, the addition of PRT was associated with a gain of 0.16 QALYs (95% CI, 0.15-0.17 QALYs) or approximately the equivalent of 2 months of perfect health, compared with ADT alone (Table 2). In addition, PRT was associated with reduced net costs of $19 472 (95% CI, $23 096-$37 362) because fewer men experienced progression. Therefore, ADT plus PRT was the dominant treatment strategy compared with ADT alone. Results were similar using the 6-weekly fraction regimen in the STAMPEDE-H trial, which was associated with $27 885 savings (95% CI, $232 272-$32 498) and gain of 0.18 QALYs (95% CI, 0.17-0.19 QALYs). With extended follow-up, PRT was associated with increased QALYs to 0.81 (95% CI, 0.73-0.89) and with reduced net costs of $30 229 (95% CI, $23 096-$37 362).

**Table 2.** Base Case Results^a^

Variable	Cost (95% CI), $	QALYs (95% CI)
37-mo Follow-up		
ADT plus PRT	114.223 (95 809 to 132 636)	1.62 (1.55 to 1.69)
PRT	94 751 (79 476 to 110 025)	1.78 (1.70 to 1.86)
Net difference	−19 472 (−16 333 to −22 611)	0.16 (0.15 to 0.17)
Lifetime follow-up		
ADT plus PRT	328 971 (251 340 to 406 602)	2.22 (2.01 to 2.43)
PRT	298 741 (228 244 to 369 238)	3.03 (2.74 to 3.32)
Net difference	−30 229 (−23 096 to −37 362)	0.81 (0.73 to 0.89)

Abbreviations: ADT, androgen deprivation therapy; PRT, prostate radiation therapy; QALYs, quality-adjusted life-years.

^a^ The 95% CIs were calculated with 10 000 individual trials.

On univariable sensitivity analysis, the model was sensitive to the HR for initial progression associated with PRT. Prostate radiation therapy was associated with improved QALYs and with reduced costs for HRs less than 0.79 (base case, 0.59). All other model parameters varied but did not significantly change the preferred strategy because no additional thresholds were encountered (eFigure 2 in the Supplement).

**Figure 2.**
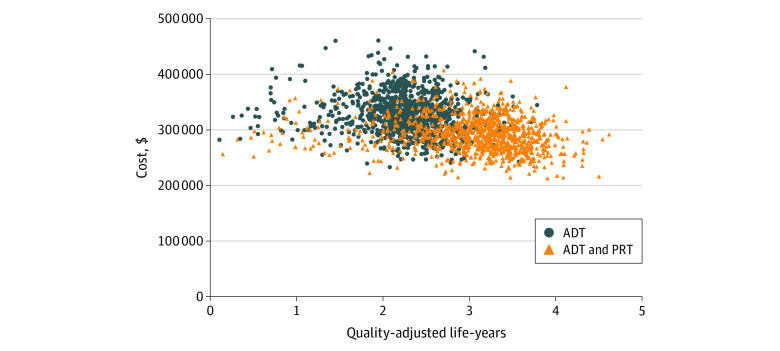
Cost-effectiveness Scatterplot of 10 000 Monte Carlo Simulations Most trials showed lower costs and higher effectiveness associated with the addition of prostate radiation therapy (PRT) to androgen deprivation therapy (ADT) compared with ADT alone.

In a sensitivity analysis, use of abiraterone at the time of diagnosis was associated with increased total costs in the ADT ($132 908; 95% CI, $111 482-$154 334) and PRT ($112 982; 95% CI, $94 768-$131 196) arms; however, the net cost savings associated with PRT were similar ($21 996; 95% CI, 18 450-$25 541), and there were gains in QALYs (0.18; 95% CI, 0.17-0.19) similar to the base case, likely reflecting similar benefits associated with abiraterone in both arms. The results were stable in probabilistic sensitivity analyses, with an expected increase in probability of cost-effectiveness with decreasing HR for progression associated with PRT and a decrease in cost associated with PRT (Table 3 and Figure 2).

**Table 3.** Summary of 1-Way and Probabilistic Sensitivity Analyses^a^

Variable	Cost (95% CI), $	QALYs (95% CI)	ICER^b^	Probability of cost-effectiveness, %
HR for progression to radiation therapy				
1.0	130 695 (100 439-160 950)	1.49 (1.20-1.78)	Dominated	0
0.75	110 309 (84 772-135 845)	1.66 (1.34-1.98)	Dominant	79
0.5	85 947 (66 050-105 843)	1.87 (1.50-2.24)	Dominant	92
Cost of PRT				
50%	86 554 (66 516-106 591)	1.78 (1.43-2.13)	Dominant	90
10%	80 275 (61 691-98 858)	1.78 (1.43-2.13)	Dominant	91
Free	78 440 (60 281-96 599)	1.78 (1.43-2.13)	Dominant	92

Abbreviations: HR, hazard ratio; ICER, incremental cost-effectiveness ratio; PRT, prostate radiation therapy; QALYs, quality-adjusted life-years.

^a^ The 95% CIs were calculated by rerunning the model 10 000 times with repeated Monte Carlo sampling with replacement from distributions of all input parameters.

^b^ A strategy was classified as dominant if it was associated with higher QALYs at lower costs than the alternative and dominated if it was associated with fewer QALYs at higher costs than the alternative.

## Discussion

After the passage of the Patient Protection and Affordable Care Act in 2010, the Centers for Medicare & Medicaid services have sought to transition from fee-for-service to value-based care, for which incentive payments are prioritized for the quality rather than quantity of health care services rendered.^25^ Cost-effectiveness analyses provide a formalized approach to determine the optimal use of health care resources to maximize benefits with regard to payer value (cost) and patient value (QALYs).^26^ We performed a CEA that evaluated the addition of PRT to systemic therapy in men with newly diagnosed low-burden mHSPC. The dominant strategy was PRT, which was associated with reduced costs by $19 472 and improved QALYs by 0.16, or approximately 2 months of perfect health gained, using the median follow-up in the STAMPEDE-H trial. We estimate larger gains (0.81 QALYs) and further reductions in net costs ($30 229) with a simulated lifetime time horizon.

To our knowledge, this is the first CEA of PRT in this setting. Sensitivity analyses around model inputs and assumptions suggest that our findings remained robust over a plausible range of values. A central finding of our analysis is that an added up-front cost of PRT may be associated with reduced net costs by improving progression-free survival in the noncurable setting by patients spending less time accumulating costs for more expensive systemic therapies. Supporting this conclusion, the model was most sensitive to the HR for progression, and PRT remained cost-effective at values less than 0.79. Our results suggest that PRT is cost-effective on the basis of data from a randomized clinical trial.

The results of our analysis contribute to a broader literature that supports the use of radiation therapy as a cost-effective treatment for cancers with limited spread or oligometastatic disease.^27,28,29,30,31^ This finding is particularly relevant in the era of focusing on value-based cancer treatments. Cancer drugs often enter the US marketplace at costs that exceed $10 000 per month and are expected to contribute to 70% of the total cost of care by 2025,^32^ highlighting the need for alternative payment systems that link the value of cancer treatments to prices. Our study supports the use of PRT as a cost-effective and of high-value treatment because it was associated with lowering payer costs and increasing patient QALYs.

### Limitations

This study has limitations. In the STAMPEDE-H clinical trial, the comparison of PRT for low-volume mHSPC was a prespecified analysis with more than 90% power for FFS and 60% power for OS based on 40% of the sample of the comparison population having low-volume disease, which was achieved in the trial. Although all criteria for a high-quality subgroup analysis were met in the STAMPEDE-H trial, this analysis was still underpowered for OS.^33^ Nonetheless, our model suggests that improvements in FFS may be cost-effective in reducing costs associated with the use of systemic therapies.

We assumed rates of toxic effects from the CHHiP trial to model more accurate long-term data because toxic effects can manifest 5 years after completion of radiation therapy, which is longer than the median follow-up time of the STAMPEDE-H trial.^10,34^ However, these rates likely overestimate toxic effects because the STAMPEDE-H trial (2.75 Gy for 20 fractions or 6 Gy for 6 fractions) used lower radiation doses than the CHHiP trial (3 Gy for 20 fractions). Therefore, this assumption potentially biases the model against PRT by using a conservative toxic effect estimate. However, we found PRT to be a dominant strategy, and varying the rate of toxic effects on sensitivity analysis did not significantly change model results. We also did not find a significant difference between 6 weekly fractions and 20 daily fractions, suggesting that either fractionation scheme may be reasonably cost-effective option.

Most cost estimates used in this study were Medicare fees with the exception of radiation therapy complication costs, which were informed by private insurance data.^21^ This finding likely overestimates the costs associated with toxic effects relative to Medicare rates because reimbursements from private plans are substantially higher. However, the model was not sensitive to fluctuations in costs associated with complications from PRT. Although the study by Pan et al^21^ provides the most granular data on intensity-modulated radiotherapy complications, a recent study^35^ of complications associated with other radiation modalities used for prostate cancer using Medicare fees found similar costs. Furthermore, costs will invariably vary across different health care systems. Costs of cancer treatment vary widely.^36,37^ In addition, although a microsimulation model is a widely accepted form of assessing cost-effectiveness, a randomized clinical trial that prospectively collects costs for comparison is the gold standard assessment.

There are limitations with regard to the systemic therapies used. Some novel therapies, such as apalutamide, were not specifically included in the cost analysis that we used.^16^ Although we are not aware of any formal CEAs of apalutamide, its price is similar to enzalumatide, and thus its omission was unlikely to impact our model results with regard to net costs.^38^ In addition, although our sensitivity analysis assessed the use of PRT when added to abiraterone, it is important to acknowledge that this warrants further investigation in a randomized clinical trial.

Utilities in our study were extracted from a study of 162 highly motivated men 60 years or older, half of whom had been diagnosed with prostate cancer.^19^ This sample may not be representative of all patients with low-volume mHSPC. However, there were no thresholds found for health state utilities in our study, suggesting patient preferences were not primary factors associated with the cost-effectiveness of PRT.

## Conclusions

For patients with newly diagnosed low-burden mHSPC, this economic evaluation supports PRT as a cost-effective treatment. The findings suggest that adjustments in the HR for progression in the STAMPEDE-H trial were associated with the cost-effectiveness of PRT. Our model was informed by high-quality data, and the addition of PRT to ADT was a dominant strategy compared with ADT alone across a wide range of assumptions. This analysis provides data to help guide the development of future clinical trials in metastatic prostate cancer to increase the value of novel cancer treatments.

## Notes

Supplement. **eTable 1.** Institutional Prostate Radiation Medicare Fees **eTable 2.** Internal Validation **eFigure 1.** Calibration of the Model Comparing Overall Survival and Failure-Free Survival to the Outcomes of the STAMPEDE-H Clinical Trial **eFigure 2.** Tornado Diagram Showing Results of All Deterministic Sensitivity Analyses
