# Errors in Abstract and Main Results

**DOI:** 10.1001/jamanetworkopen.2021.0615

**Published:** 2021-02-08

In the Original Investigation titled “Cost-effectiveness of Prostate Radiation Therapy for Men With Newly Diagnosed Low-Burden Metastatic Prostate Cancer,”^1^ published January 13, 2021, in the abstract results and the first paragraph of the main results section, the 95% CI for the reduction in net costs of $19 472 was changed to $16 333 to $22 611. Also, in the first paragraph of the main results, the 95% CI for the savings of $27 885 was changed to $23 272 to $32 498. This article has been corrected.
