# Supplementary material for: Cost-effectiveness of Prostate Radiation Therapy for Men With Newly Diagnosed Low-Burden Metastatic Prostate Cancer
Source: JAMA Netw Open. 2021 Jan 13;4(1):e2033787. doi: 10.1001/jamanetworkopen.2020.33787 (PMC7807293; doi:10.1001/jamanetworkopen.2020.33787)
Supplement: Supplement. — eTable 1. Institutional Prostate Radiation Medicare Fees eTable 2. Internal Validation eFigure 1. Calibration of the Model Comparing Overall Survival and Failure-Free Survival to the Outcomes of the STAMPEDE-H Clinical Trial eFigure 2. Tornado Diagram Showing Results of All Deterministic Sensitivity Analyses [file jamanetwopen-e2033787-s001.pdf]

Supplementary Online Content

Lester-Coll NH, Ades S, Yu JB, Atherly A, Wallace HJ, Sprague BL. Cost-effectiveness of prostate radiation therapy in men with newly diagnosed low-burden metastatic prostate cancer. *JAMA Netw Open*. 2021;4(31):e2033787. doi:10.1001/jamanetworkopen.2020.33787

eTable 1. Institutional Prostate Radiation Medicare Fees

eTable 2. Internal Validation

eFigure 1. Calibration of the Model Comparing Overall Survival and Failure-Free Survival to the Outcomes of the STAMPEDE-H Clinical Trial

eFigure 2. Tornado Diagram Showing Results of All Deterministic Sensitivity Analyses

This supplementary material has been provided by the authors to give readers additional information about their work.

© 2021 Lester-Coll NH et al. *JAMA Network Open.*

eTable 1. Institutional Prostate Radiation Medicare Fees

	CPT code	Payment rate per unit MPFS	Payment rate per unit OPPS			
**Interstitial fiducial device**	55876	$105.12	$1,186.68			
US guidance for fiducial insertion	76942	$32.08	$0.00			
Treatment Planning	77263	$172.30				
Dosimetry Calculations	77300	$33.52	$125.35			
IMRT planning	77301	$429.67	$1,186.68			
Physics weekly check	77336		$125.35			
IMRT MLC	77338	$230.70	$323.09			
IMRT Simple	77385		$522.31			
Fiducial Marker	A4648		$0.00			
Ultrasound guidance	G6001	$32.08				
Weekly on treatment management	77427	$191.16				
**44 fractions**		**Pro units**		**Tech units**		
Interstitial fiducial device	55876	1	$105.12	1	$1,186.68	
US guidance for fiducial insertion	76942	1	$32.08	1	$0.00	
Treatment Planning	77263	1	$172.30	0		
Dosimetry Calculations	77300	5	$167.60	5	$626.75	
IMRT planning	77301	1	$429.67	1	$1,186.68	
Physics weekly check	77336	0		9	$1,128.15	
IMRT MLC	77338	1	$230.70	1	$323.09	
IMRT Simple	77385	0		44	$22,981.64	
Fiducial Marker	A4648	0		2	$0.00	
Ultrasound guidance	G6001	44	$1,411.52	0		
Weekly on treatment management	77427	9	$1,720.44			
Total Medicare Fee			$4,269.43		$27,432.99	$31,702.42
**20 fractions**		**Pro units**		**Tech units**		
Interstitial fiducial device	55876	1	$105.12	1	$1,186.68	
US guidance for fiducial insertion	76942	1	$32.08	1	$0.00	
Treatment Planning	77263	1	$172.30	0		
Dosimetry Calculations	77300	2	$67.04	2	$250.70	
IMRT planning	77301	1	$429.67	1	$1,186.68	
Physics weekly check	77336	0		4	$501.40	
IMRT MLC	77338	1	$230.70	1	$323.09	
IMRT Simple	77385	0		20	$10,446.20	
Fiducial Marker	A4648	0		2	$0.00	
Ultrasound guidance	G6001	20	$641.60	0		
Weekly on treatment management	77427	4	$764.64			
Total Medicare Fee			$2,443.15		$13,894.75	$16,337.90

© 2021 Lester-Coll NH et al. *JAMA Network Open.*

**6 fractions**		**Pro units**		**Tech units**		
Interstitial fiducial device	55876	1	$105.12	1	$1,186.68	
US guidance for fiducial insertion	76942	1	$32.08	1	$0.00	
Treatment Planning	77263	1	$172.30	0		
Dosimetry Calculations	77300	2	$67.04	2	$250.70	
IMRT planning	77301	1	$429.67	1	$1,186.68	
Physics weekly check	77336	0		6	$752.10	
IMRT MLC	77338	1	$230.70	1	$323.09	
IMRT Simple	77385	0		6	$3,133.86	
Fiducial Marker	A4648	0		2	$0.00	
Ultrasound guidance	G6001	20	$641.60	0		
Weekly on treatment management	77427	6	$1,146.96			
Total Medicare Fee			$2,825.47		$6,833.11	$9,658.58

Abbreviations: IMRT, intensity-modulated radiation therapy; MLC, multileaf collimator; US, ultrasound; MPFS, Medicare Physician Fee Schedule; OPPS, Outpatient Prospective Payment System

© 2021 Lester-Coll NH et al. *JAMA Network Open.*

eTable 2. Internal Validation

Internal validation metric	Model	Clinical Data
3-year overall survival ADT	72%	73%
3-year overall survival PRT	79%	81%
3-year failure-free survival ADT	33%	30%
3-year failure-free survival ADT PRT	48%	50%
5-year grade 2+ GI toxicity	12%	12%
5-year grade 2+ GU toxicity	12%	12%

Abbreviations: ADT, androgen deprivation therapy; PRT, prostate radiation therapy; GI, gastrointestinal; GU, genitourinary

© 2021 Lester-Coll NH et al. *JAMA Network Open.*

eFigure 1. Calibration of the Model Comparing Overall Survival and Failure-Free Survival to the Outcomes of the STAMPEDE-H Clinical Trial

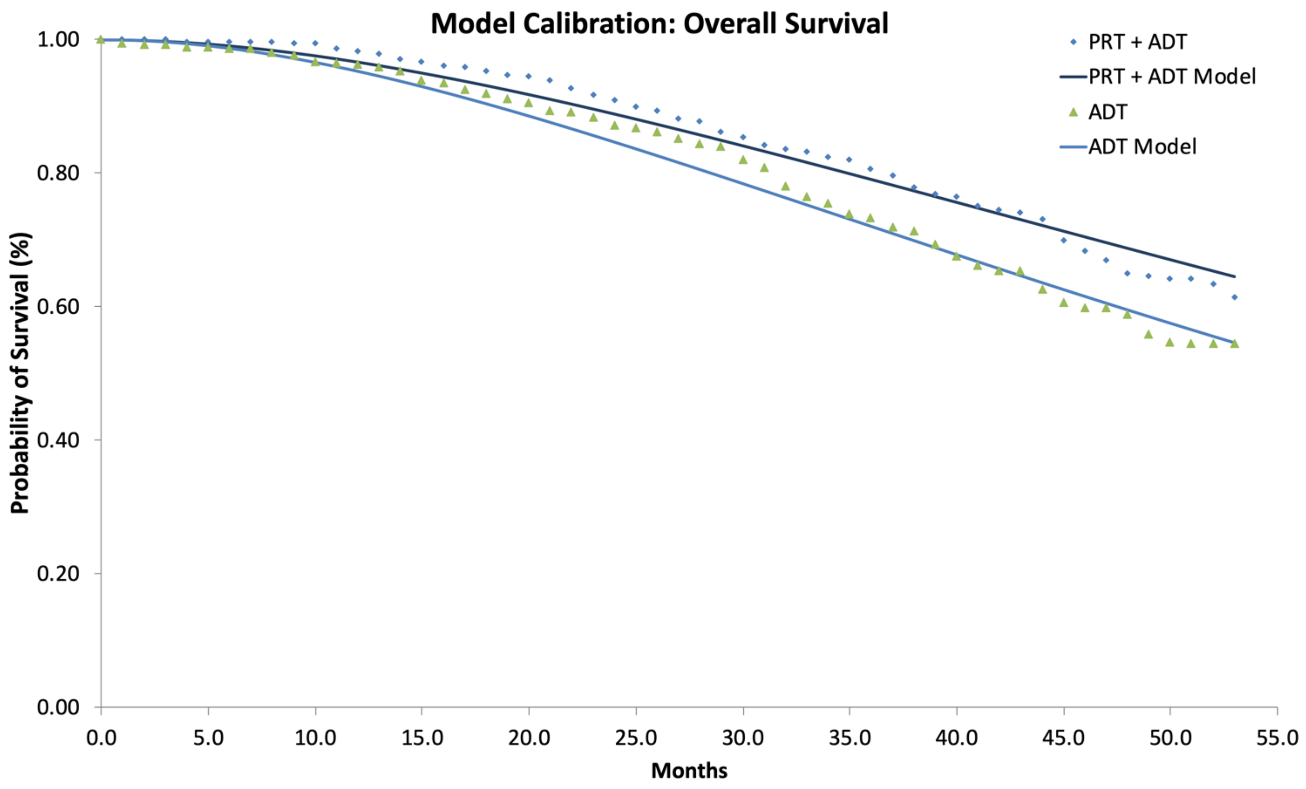

© 2021 Lester-Coll NH et al. *JAMA Network Open.*

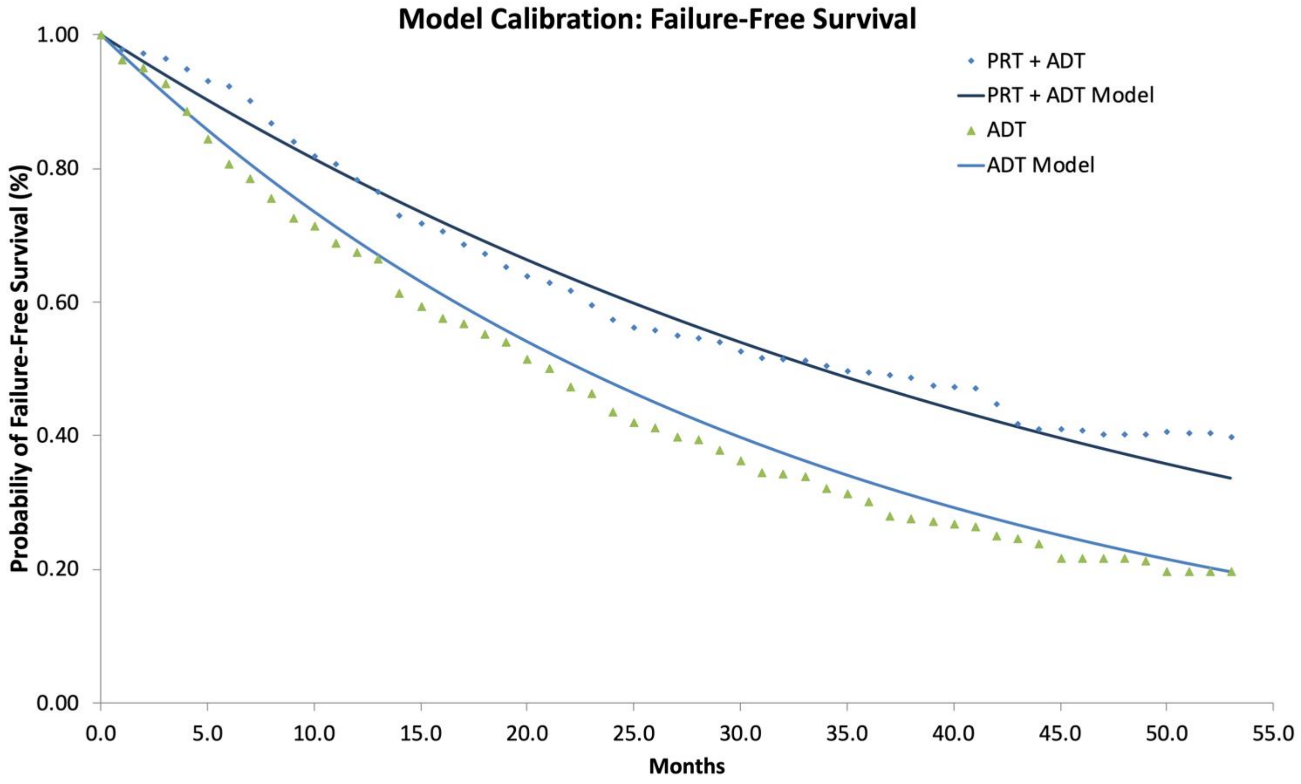

© 2021 Lester-Coll NH et al. *JAMA Network Open.*

eFigure 2. Tornado Diagram Showing Results of All Deterministic Sensitivity Analyses

A vertical black bar signifies a significant threshold where the preferred strategy changed. This was only encountered for the hazard ratio for progression with PRT.

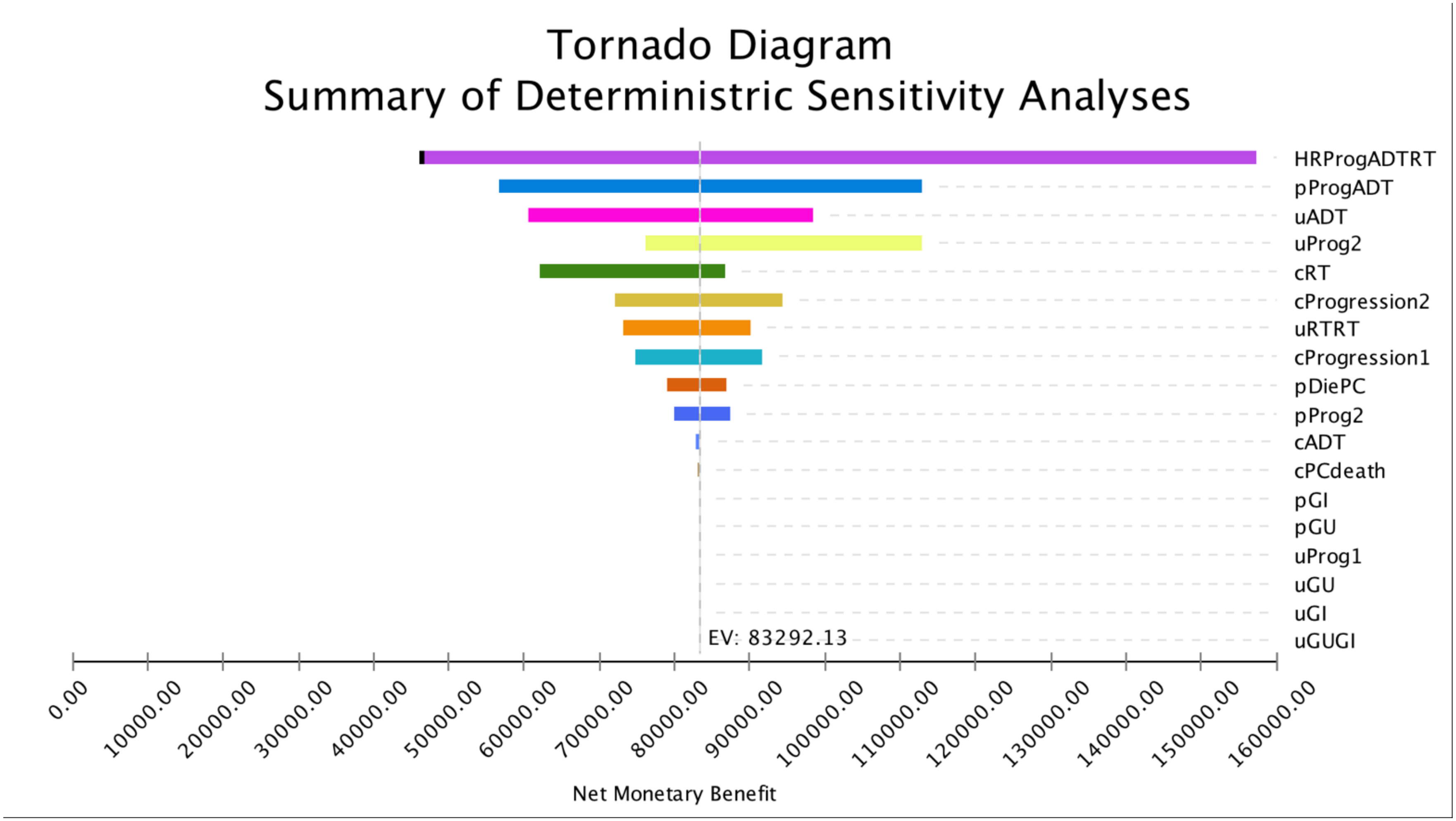

© 2021 Lester-Coll NH et al. *JAMA Network Open.*
